# Comprehensive investigation of ergonomic challenges and predictors of work-related musculoskeletal disorders among intensive care unit nurses of Western India through convergent mixed methods study

**DOI:** 10.1186/s12891-025-08379-4

**Published:** 2025-02-07

**Authors:** Sonali Detroja, Rajkumar Mahajan, Ankit Sheth

**Affiliations:** 1 Community Medicine Department, B J Medical College and Civil Hospital, Ahmedabad, India; 2 ICMR– National Institute of Occupational Health, Ahmedabad, India

**Keywords:** Musculoskeletal pain, Intensive care units, Nurses, Ergonomics, Workplace

## Abstract

**Background:**

Work-related musculoskeletal disorders (WRMSDs) are significant concern in intensive care units (ICUs) due to distinct work environment. This study aims to comprehensively investigate determinants of WRMSDs and ergonomic challenges, specific to ICU nurses, providing valuable insights for targeted interventions.

**Methods:**

In this six-month convergent mixed methods study, 200 consenting nurses with over one year of experience from seven ICUs across three public tertiary hospitals of Ahmedabad participated. Structured questionnaires were used to collect data on socio-demographics, occupation, physical and workplace factors, risk perception, and musculoskeletal pain (through modified Nordic Musculoskeletal Questionnaire) and ergonomic issues using REBA (Rapid Entire Body Assessment) scale. Qualitative insights were obtained through in-depth interviews until saturation of responses. Statistical analysis involved chi-square, t-test and logistic regression, with a significance level set at *p* < 0.05. Thematic analysis was used to interpret the findings of qualitative study.

**Results:**

The study included predominantly female ICU nurses (78%) with a mean age of 34 years. A high prevalence of WRMSDs (84%) was observed, with the lower back and neck being the most affected regions. Nurses with WRMSDs reported significantly higher mean scores for physical factors (15.1 vs. 11.1, *p* = 0.00), physical workload (12.8 vs. 10.7, *p* = 0.001), work environment (13.1 vs. 10.1, *p* = 0.00), and risk perception (11.8 vs. 8.9, *p* = 0.00) compared to those without WRMSDs. Logistic regression identified key risk factors for WRMSDs, including longer ICU tenure [OR = 1.4 (1.13–1.66)], high shift frequency [OR = 2.7 (1.27–5.95)], higher physical factor score [OR = 1.2 (1.05–1.42)], higher physical workload score [OR = 1.2 (1.09–1.49)], higher risk perception [OR = 1.3 (1.10–1.78)], and lack of exercise [OR = 0.5 (0.27–0.93)]. The qualitative findings highlighted key ergonomic challenges, including inadequate equipment, heavy patient loads, poor posture during tasks, and insufficient breaks, contributing to WRMSDs among ICU nurses.

**Conclusion:**

The findings underscore urgent need for targeted interventions to address risk factors associated with WRMSDs, including ergonomic training, workplace modifications, and education programs to enhance risk awareness and preventive behaviours. Future research should focus on developing and evaluating comprehensive interventions that integrate both physical and work environment factors to effectively mitigate WRMSDs among ICU nurses.

## Introduction

Work-related musculoskeletal disorders (WRMSDs) are a significant occupational health concern, characterized by conditions where the work environment and job tasks substantially contribute to the development, persistence, or exacerbation of musculoskeletal symptoms [1]. These disorders commonly affect muscles, tendons, intervertebral discs, nerves, joints, and cartilage, leading to debilitating symptoms such as lower back pain, neck discomfort, and shoulder pain [2, 3]. These disorders arise due to various factors inherent to the nature of the work including prolonged duty hours, demanding tasks, repetitive movements and unsafe working conditions [4, 5, 6]. 

For nurses, WRMSDs are a significant occupational health problem, with a substantial global prevalence ranging from 40 to 85% annually, as reported in studies from countries such as Estonia, Nigeria, Uganda, California, Brazil and Italy [2, 7, 10]. Similarly, in China, WRMDs among nurses have been reported to have a prevalence ranging from 56 to 92% [11, 13]. These disorders are particularly prevalent among nurses working in general healthcare setting due to the physical demands of their work. The management of WRMSDs involves a variety of strategies, including education, ergonomic interventions, and on-site treatment programs, which are often more effective when combined with stretching and strengthening exercises targeting muscles prone to shortening and hypertonicity [14, 15]. 

In intensive care units (ICUs), nurses face unique ergonomic challenges that are distinct from those encountered in general healthcare settings. ICU patients are often immobile, requiring frequent and complex patient handling tasks such as repositioning and transferring, which place significant strain on nurses [16, 18]. The ICU setting also demands prolonged periods of standing, repetitive movements, and high levels of physical exertion [16, 18]. In resource-constrained settings like India, where high patient loads and staff shortages exacerbate these challenges, nurses working in ICUs are at an elevated risk of WRMSDs [19]. 

Despite extensive research on estimating prevalence of WRMSDs among nurses in general, significant gaps remain in understanding the unique challenges and risks faced by nurses in ICU settings. Existing studies predominantly focus on general nursing populations, often overlooking comprehensive assessment of ICU-specific risk factors such as the physical strain of handling critically ill, immobile patients, environmental conditions, and the absence of ergonomic training or equipment [7, 13]. 

Given the critical role ICU nurses play in delivering care to critically ill patients, it is imperative to address the above gaps in the literature. This study aims to investigate the prevalence of WRMSDs among ICU nurses in India, identify associated work-related and environmental factors, and explore perceived ergonomic challenges and coping mechanisms within the ICU setting. By addressing these gaps, this study seeks to provide evidence to inform the development of targeted interventions and occupational health policies that mitigate WRMSDs among ICU nurses.

We hypothesize that ICU nurses experience a higher prevalence of WRMSDs, driven by unique work-related factor and challenges in these settings.

## Methods

### Study design and setting

This cross-sectional study was conducted over six months in Ahmedabad city, Gujarat, India, to evaluate musculoskeletal pain and its associated factors among nurses working in ICUs of three largest public tertiary care hospitals. These ICUs admitted critically ill patients from various hospital departments. The study employed a convergent mixed-methods approach, where quantitative and qualitative data were collected and analysed simultaneously, with findings integrated during interpretation to provide a comprehensive understanding of WRMSDs. The design adhered to principles outlined by Creswell and Clark for mixed-methods research [20], followed the Good Reporting of a Mixed Methods Study (GRAMMS) criteria [21], and adhered to the STROBE (Strengthening the Reporting of Observational Studies in Epidemiology) guidelines for cross-sectional reporting [22]. 

### Study population

The study population comprised nurses working in the ICUs of public tertiary care hospitals in Gujarat, India. These nurses were selected due to the physically demanding nature of their roles, which often puts them at a higher risk of developing WRMSDs. Inclusion criteria required nurses to be registered professionals with at least one year of ICU experience and to be involved in direct patient care duties on a daily basis. Nurses with pre-existing comorbidities such as diabetes, autoimmune diseases such as rheumatoid arthritis, or gout, which could independently contribute to musculoskeletal symptoms were excluded from this study. Additionally, those with recent viral infections causing myalgia, non-occupational trauma leading to musculoskeletal injuries, or those on extended medical leave were also excluded to minimize potential confounding factors.

### Sample size and sampling strategy

Considering an estimated 85% prevalence of musculoskeletal disorders (MSDs) among nurses, as reported in previous study [23], the sample size was determined to be 196, rounded to 200, to estimate the expected proportion with 5% absolute precision and 95% confidence, using Open Epi Version 3.01.

A two-stage simple random sampling approach was adopted to select the study sample. In the first stage, the three largest public tertiary care hospitals in Ahmedabad city were identified, and a comprehensive list of their ICUs, excluding neonatal ICUs was obtained. From this pool of fourteen ICUs, seven ICUs were randomly selected using a random number generator, ensuring unbiased and representative selection of units. In the second stage, a complete list of all eligible nurses working in these seven ICUs was compiled. Each nurse meeting the inclusion and exclusion criteria was assigned a unique identification number, and 200 nurses were randomly selected using a random number generator. Proportional allocation was applied to ensure that the number of nurses selected from each ICU corresponded to the size of the nursing workforce in that unit.

### Data collection tools

A structured questionnaire was used to capture sociodemographic details (age, sex, marital status, and highest education level) and occupational information, including years of experience, job title, duty hours, and frequency of breaks.

Physical factors, physical workload, work environment, and risk perception were assessed using a customised five-point Likert scale questionnaire specifically tailored for this study, drawing upon constructs reported in the existing literature [24, 25]. The internal consistency (Cronbach’s alpha) for these domains was determined to be 0.846, 0.867, 0.878, and 0.823, respectively. Content validity for the tool, assessed by three independent experts in the field, was found to have a Content Validity Index (CVI) of 1, indicating alignment of the items with the intended constructs.

The modified Nordic Musculoskeletal Questionnaire (NMQ) was utilized to locate musculoskeletal pain sites and assess pain severity among participants [26]. This tool, which is freely available, was adapted for this study by excluding certain items that were deemed less relevant to the study objectives. Despite these modifications, the internal consistency of the revised tool (Cronbach’s alpha = 0.885) demonstrated high reliability. All three subject experts endorsed modified NMQ as valid (CVI = 0.95). The original version of the NMQ has been extensively validated and is widely recognized for its robustness in assessing musculoskeletal symptoms.

The Rapid Entire Body Assessment (REBA) scale [27] was employed to evaluate the ergonomic challenges associated with specific tasks performed by participants, thereby assessing their potential contribution to the development of WRMSDs. The REBA scale demonstrated acceptable internal consistency (Cronbach’s alpha of 0.818) and effective inter-observer reliability (Fleiss kappa = 0.61).

To ensure clarity and comprehensiveness, all tools were pre-tested with 10 participants prior to the actual data collection. This pre-testing phase allowed for refinement of the tools to address any issues related to feasibility or participant understanding, thereby enhancing the reliability of the data collection process.

Qualitative data were collected through in-depth interviews to gain deeper insights into the challenges faced by nurses in ICUs, their coping mechanisms, institutional support, and potential preventive measures for WRMSDs. Interviews and discussions continued until saturation of responses was achieved, ensuring a comprehensive understanding of participants’ perspectives.

The questionnaires were administered in person by the investigator, ensuring clarity in the instructions and addressing any participant queries. The participants completed the questionnaires taking approximately 30 min to respond. The qualitative surveys for limited participants took an additional 30 min to complete. Data collection was conducted by the primary author, an M.D. doctor, with the assistance of a subject expert in occupational health and qualitative research, using physical forms filled out by participants under supervision.

### Study variables and measures

The primary dependent variable was musculoskeletal symptoms, defined as symptoms lasting ≥ 1 week, occurring ≥ 1 per month, of at least moderate intensity, and attributed to work-related events. The symptoms were measured using modified NMQ in which diagram of the body was depicted to help participants accurately locate the region and duration of pain by author herself. Participants were then asked to rate the intensity of their pain on a Likert scale (1 = excruciating, 2 = severe, 3 = moderate, 4 = mild), describe the type of pain experienced, and indicate whether they sought medical intervention for symptom management.

Independent variables included demographics, physical factors, job and workplace-related factors, environmental factors, and risk perception.

*Demographic details* included age, sex, marital status and highest education level. *Occupational details* comprised years of experience, job title, and specifics regarding duty hours, breaks, and frequency of patient handling activities. *Personal habits*, such as smoking status, duration, and daily cigarette consumption, were also recorded.

*Physical factors* were assessed through a series of five-point Likert scale-based questions (1 = never to 5 = very often) regarding the frequency of patient handling tasks, lifting and transferring patients, utilization of lifting aids, and assistance from colleagues during patient shifting from wheelchair to bed and vice versa. Additionally, questions regarding continuous standing, repetitive bending, heavy lifting (> 20 kg), and the frequency of performing repetitive movements were included to gauge the *physical workload/ demand* experienced by participants.

*Environmental factors and risk perception* were evaluated through inquiries regarding ergonomic training, patient handling techniques, and comfort level in reporting issues to superiors. Engagement of the participants in stretching and exercise activities, sleep disturbances, perceptions of work-related pain causation, and disruptions in personal life due to work were assessed using five-point Likert scale responses (1 = never/strongly disagree to 5 = very often/strongly agree).

*REBA* scale provides a single final score based on the posture evaluated, force requirement, type of movement, frequency of movement and coupling observed within the task. The methods and forms used for REBA are widely accessible in academic literature. The REBA score categorizes the risk into five levels: negligible (score 1), low (scores 2–3), medium (scores 4–7), high (scores 8–10), and very high (scores 11–15).

### Data analysis

Quantitative data were analysed using licensed SPSS 26.0. Descriptive statistics, including frequencies, percentages, means, and standard deviations, were calculated to summarize the data. The mean scores for physical factors, physical workload, work environment, and risk perception were analysed using Student’s t-tests to determine significant differences between the groups with and without WRMSDs. Chi-square tests were used to examine associations between categorical variables of demographic characteristics, job-related factors, and the presence of WRMSDs. A logistic regression model was used to identify the predictors of WRMSDs. Significance level for all inferential statistical tests was set at 0.05. Qualitative data were assigned codes and categories and analysed thematically to identify patterns, themes, and key insights emerging from interviews.

## Results

### Quantitative study findings

This study included 200 ICU nurses across seven different ICU in three hospitals of Gujarat. The demographic and occupational characteristics of ICU nurses were examined in relation to the prevalence of WRMSDs. Among the 200 participants, 168 (84%) reported experiencing WRMSDs, while 32 (16%) did not. There were no significant differences observed in the distribution of age (*p* = 0.56), sex (*p* = 0.65), or education level (*p* = 0.29) between the two groups. However, significant associations were found between WRMSDs and marital status (*p* = 0.003), years working in the ICU (*p* = 0.000), ICU shift frequency (*p* = 0.000), number of breaks per shift (*p* = 0.009), exercise frequency (*p* = 0.01), and current smoking status (*p* = 0.02) (Table 1).

**Table 1:** Demographics and occupational details of ICU nurses and its association with work-related musculoskeletal disorders (WRMSDs)

Variables	WRMSDs		Significance
	Yes (*n* = 168)	No (*n* = 32)	
Age in years, n (%)			
18–30	60 (35.7)	13 (40.6)	*p* = 0.56
31–40	80 (47.6)	16 (50.0)	
> 40	28 (16.7)	3 (9.4)	
Sex, n (%)			
Male	36 (21.4)	8 (25.0)	*p* = 0.65
Female	132 (78.6)	24 (75.0)	
Education, n (%)			
Higher secondary/ diploma	80 (47.6)	12 (37.5)	*p* = 0.29
Graduate and beyond	88 (52.4)	20 (62.5)	
Marital status, n (%)			
Unmarried/ Divorce/ separated	66 (39.3)	4 (12.5)	***p*** **= 0.003**
Married	102 (60.7)	28 (87.5)	
Years working in ICU, n (%)			
1–5	60 (35.7)	24 (75.0)	***p*** **= 0.000**
6–10	64 (38.1)	8 (25.0)	
> 10	44 (26.2)	0 (0)	
ICU shift frequency (days per week), n (%)			
≤ 4	56 (33.4)	22 (68.8)	***p*** **= 0.000**
5–7	112 (66.6)	10 (31.2)	
Length of breaks, minutes, n (%)			
≤ 10	114	26	*p* = 0.12
10–30	54	6	
Number of breaks per shift, n (%)			
≤ 1	48 (28.6)	2 (6.2)	***p*** **= 0.009**
2	48 (28.6)	16 (50.0)	
3 or more	72 (42.8)	14 (43.8)	
Exercise, n (%)			
Never	60 (35.7)	2 (6.2)	***p*** **= 0.01**
Seldom (< 5 days/ month)	56 (33.3)	6 (18.8)	
Occasional (6–10 days/ month)	24 (14.3)	8 (25.0)	
Often (> 10 days/month)	28 (16.7)	8 (25.0)	
Current smoking status, n (%)			
Currently non-smoker	114 (67.9)	28 (87.5)	***p*** **= 0.02**
Currently smoker	54 (32.1)	4 (12.5)	

**Fig. 1:**
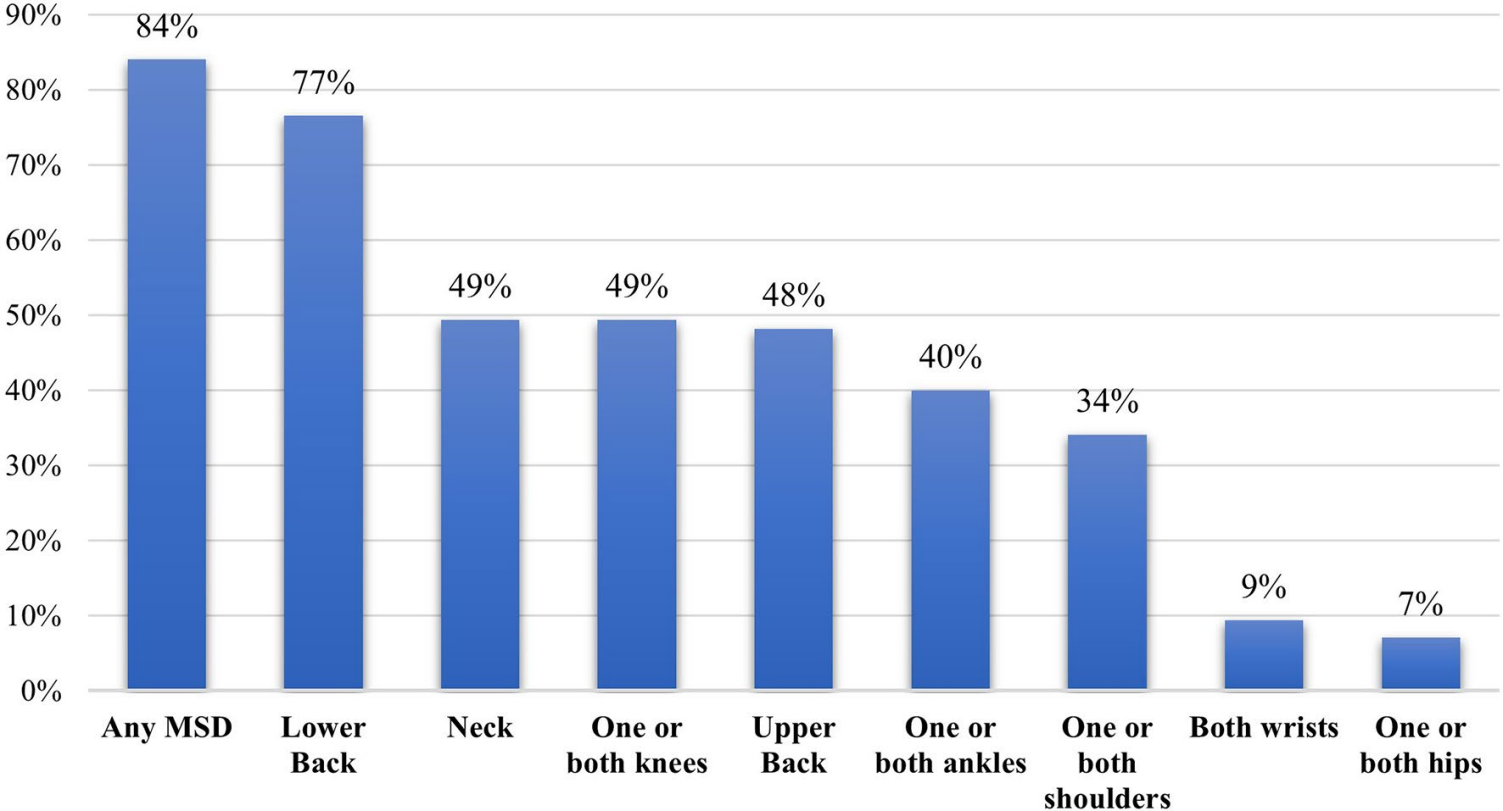
Prevalence of work-related musculoskeletal disorders (WRMSDs) according to body region among ICU nurses reported by modified Nordic Musculoskeletal Questionnaire

Low back pain was the most commonly reported WRMD (77%), followed by neck pain (49%), knee pain (49%) and upper back pain (48%) (Fig. 1). The majority of participants with WRMSDs reported experiencing dull ache-type pain (85.9%), with moderate to severe pain intensity being more prevalent. Among those seeking intervention, medication was more commonly sought than physiotherapy (Table 2).

**Table 2:** Type of pain, intensity of pain and intervention sought by work-related musculoskeletal disorders (WRMSDs) positive participants (*n* = 168)

Variables	No. of participants, *n* (%) [a]	On medications, *n* (% of a)	On physiotherapy, *n* (% of a)
**Type of pain**			
Dull ache	144 (85.9)	88 (61.1)	42 (29.1)
Sharp pain	24 (14.1)	16 (66.6)	10 (41.6)
**Intensity of pain**			
Mild	10 (6)	0 (0)	0 (0)
Moderate	76 (45.2)	36 (47.3)	14 (18.4)
Severe	66 (39.3)	52 (78.8)	28 (42.4)
Excruciating	16 (9.5)	16 (100)	10 (62.5)

Participants with WRMSDs reported significantly higher mean scores for physical factors (*p* = 0.000), physical workload (*p* = 0.001), work environment (*p* = 0.000), and risk perception (*p* = 0.000) compared to those without WRMSDs (Table 3).

**Table 3:** Association between physical factors, physical workload, work environment and risk perception with work-related musculoskeletal disorders (WRMSDs)

Variables	WRMSDs		Significance
	Yes (*n* = 168)	No (*n* = 32)	
Physical factors (total score 50), mean (SD)	15.1 (6.5)	11.1 (2.0)	*p* = 0.000
Physical workload (total score 20), mean (SD)	12.8 (2.4)	10.7 (1.5)	*p* = 0.001
Work environment (total score 20), mean (SD)	13.1 (3.4)	10.1 (2.7)	*p* = 0.000
Risk perception (total score 20), mean (SD)	11.8 (3.2)	8.9 (3)	*p* = 0.000

The logistic regression analysis revealed several significant risk factors associated with the prevalence of WRMSDs among ICU nurses. Participants with longer tenure in the ICU (OR = 1.4, 95% CI: 1.13–1.66, *p* = 0.001), working 5–7 days per week (OR = 2.7, 95% CI: 1.27–5.95, *p* = 0.003), higher physical factor score (OR = 1.2, 95% CI: 1.05–1.42, *p* = 0.009), higher physical workload score (OR = 1.2, 95% CI: 1.09–1.49, *p* = 0.04), higher risk perception (OR = 1.3, 95% CI: 1.10–1.78, *p* = 0.01), and those never exercising (OR = 0.5, 95% CI: 0.27–0.93, *p* = 0.02) were more likely to report WRMSDs. The logistic regression model was statistically significant and the model explained 58% (Nagelkerke R^2^) of the variance (Table 4).

**Table 4:** Logistic regression analysis to determine the risk factors of work-related musculoskeletal disorders (WRMSDs)

Risk factors	OR (95% CI)	Significance
Marital status	0.7 (0.33–1.58)	0.42
Smoking status	1.5 (0.72–3.34)	0.26
Years working in ICU	1.4 (1.13–1.66)	**0.001**
Shift frequency in ICU	2.7 (1.27–5.95)	**0.003**
Number of breaks per shift	0.5 (0.20–1.20)	0.12
Exercise	0.5 (0.27–0.93)	**0.02**
Physical factors	1.2 (1.05–1.42)	**0.009**
Physical workload	1.2 (1.09–1.49)	**0.04**
Work environment	1.0 (0.45–2.30)	0.95
Risk perception	1.3 (1.10–1.78)	**0.01**

The REBA scores were used to evaluate ergonomic risk levels. Among the 200 participants, 50 individuals (25%) were categorized in the negligible risk category, 32 (16%) in the low risk category, 51 (25.5%) in the medium risk category, 49 (24.5%) in the high risk category, and 18 (9%) in the very high risk category. These findings indicate that a significant portion of the participants are at medium to very high ergonomic risk, highlighting the need for targeted interventions to address musculoskeletal disorder risks among ICU nurses (Table 5).

**Table 5:** Rapid entire body Assessment (REBA) score to assess the ergonomic risk level among ICU nurses

REBA score	No. of participants	Risk level	Action needed
1	50	Negligible	None necessary
2–3	32	Low	May be necessary
4–7	51	Medium	Necessary
8–10	49	High	Necessary soon
11–15	18	Very high	**Necessary Now**

### Qualitative study findings

The median work experience of the fourteen in-depth interviewees, was 7 years. The analysis of the codes and categories from the transcript revealed four primary themes based on the responses of participants: the challenges faced in ICU, coping mechanisms, institutional support and preventive measures.

Challenges faced in ICU: Participants commonly mentioned challenges related to heavy patient loads, frequent patient handling, long working hours, and the physical demands of their tasks. They also discussed the emotional toll of caring for critically ill patients and the need for emotional support.

- *“The constant rush*,* attending to one patient after another*,* without much time to rest in between*,* it really takes a toll on your body.”*
- *“Standing for long hours during procedures is exhausting. Sometimes*,* it feels like there’s no end to it.”*
- *“The emotional burden is immense*,* especially when patients are critical. It adds to the physical strain we already face.”*

Coping mechanisms: Nurses described various coping strategies, including seeking social support from colleagues, practicing mindfulness or relaxation techniques during breaks, and finding satisfaction in providing quality patient care despite the challenges.

- *“Talking to my colleagues helps a lot. We share our experiences and have a laugh about it.”*
- *“Knowing that I’m making a difference in patients’ lives keeps me going. Despite the challenges*,* it’s rewarding to see them recover.”*

Institutional support: Participants highlighted the importance of institutional support in addressing WRMSDs. They emphasized the need for ergonomic training, access to ergonomic equipment, and clear communication channels for reporting workplace hazards or injuries. Some also expressed the need for counselling services to address the emotional stress of their work.

- *“We need better training on how to handle patients safely. Patient handling should be ergonomic so that our backs do not hurt.”*
- *“We need more ergonomic equipment. This would make a big difference.”*
- *“There should be someone we can talk to about the stress. It’s not just about physical health; our mental well-being matters too.”*

Potential preventive measures discussed by participants included implementing ergonomic practices such as proper lifting techniques, adjusting work schedules to allow for adequate rest and recovery, providing regular breaks, and promoting physical exercise or stretching routines.

- *“We need more breaks during shifts. Even just a few minutes to stretch and move around can make a difference.”*
- *“Proper lifting techniques are crucial. We often lift heavy patients without realizing the strain it puts on our bodies.”*
- *“Encouraging regular exercise and providing resources for it would be beneficial. It’s hard to find time for self-care outside of work.”*

## Discussion

This convergent mixed-method study investigated the prevalence of WRMSDs among 200 ICU nurses in public tertiary care hospitals in Gujarat and the risk factors associated with WRMSDs. The prevalence of WRMSDs among ICU nurses was 84%, with low back (77%) and neck (49%) as the most affected regions. These rates are notably higher than the reports from countries such as Brazil, Italy, India, Nigeria, Ethiopia, and Saudi Arabia, which involved general nursing populations [10, 28, 29, 30, 31]. Studies focusing on ICU nurses have reported consistent or higher prevalence rates, highlighting the ergonomic and workload challenges associated with critical care environments [24, 29, 32, 33]. 

In this study, several factors were independently identified as significantly associated with WRMSDs, including married status, longer ICU tenure, high shift frequency, fewer breaks per shift, absence of exercise, and smoking habits.

Previous evidence highlights that unmarried nurses report a higher prevalence of WRMSDs, potentially due to the absence of familial support to buffer the effects of occupational stress [34, 35]. Longer ICU tenure has been associated with increased WRMSD prevalence, likely due to prolonged exposure to ergonomic risks and age-related muscle strength decline [7, 19]. Research from China underscores that fewer breaks, uncomfortable break settings, and lack of exercise contribute to higher WRMSD prevalence [24]. Nurses with lower shift frequencies and more breaks may have sufficient time for recovery, potentially resulting in a lower WRMSDs [24]. Additionally, tobacco use has been identified as a modifiable risk factor for WRMSDs, highlighting its importance as a target for prevention strategies [36]. 

The participants with WRMSDs in our study exhibited significantly higher mean scores for physical factors, physical workload, work environment, and risk perception compared to those without WRMSDs. The association between physical risk factors and WRMSDs has been supported by previous research, suggesting that nurses exposed to high physical demands are more susceptible to WRMSDs [37, 39]. Risk perception, though a relatively underexplored factor, emerged as a significant modifiable determinant. Studies suggest that awareness of occupational hazards can motivate healthy behaviours, highlighting the need for education and training interventions to enhance risk awareness and reduce WRMSDs [25, 40, 42]. Additionally, the role of workplace safety and environmental conditions is critical, as unsafe environments not only exacerbate physical strain but also influence nurses’ motivation and performance. [41–42] Organizational efforts to foster a safety culture and provide ergonomic training could mitigate these risks.

Logistic regression analysis identified several significant predictors of WRMSDs, including ICU tenure, frequent shifts, absence of exercise, and high physical workload and risk perception scores. These findings underscore the importance of targeted interventions addressing modifiable risk factors, such as promoting regular exercise, optimizing shift patterns, and implementing ergonomic improvements in ICU settings.

The qualitative interviews complemented the quantitative findings, highlighting ICU nurses’ challenges, such as heavy patient loads, prolonged duty hours, and emotional stress. Coping mechanisms, including social support and job satisfaction, underscore the resilience of nurses, while institutional support in the form of ergonomic training and proper equipment was recognized as essential for addressing WRMSDs. Preventive measures, such as promoting physical exercise and ergonomic practices, were emphasized by participants, further reinforcing the need for a holistic approach to mitigating WRMSDs.

The study has several strengths, including its mixed-method design, which provided a comprehensive understanding of WRMSDs among ICU nurses. The use of validated tools ensured robust assessments of musculoskeletal health and risk factors. Moreover, the focus on ICU nurses, a population often overlooked in WRMSD research, adds novelty to the study.

However, the study is not without limitations. First, the cross-sectional design precludes causal inferences. Second, the reliance on self-reported data may introduce reporting biases, although efforts were made to mitigate this through rapport-building and clarification of questions to encourage truthful reporting. Third, the findings are specific to public tertiary hospitals in Gujarat and may not be generalizable to other healthcare settings or regions. Future research should address these limitations through longitudinal designs and broader, more diverse sample populations.

### Implications for clinicians and research

The findings have several implications for clinical practice and research. For clinicians, the study highlights the need for regular ergonomic training, promotion of physical exercise, and ensuring adequate recovery time during shifts to prevent WRMSDs. Hospital administrators should prioritize the implementation of workplace interventions, such as modifying work schedules, optimizing staffing, and improving access to ergonomic tools and equipment, to mitigate physical strain on ICU nurses. Furthermore, fostering a culture of safety and risk awareness through targeted education programs can mitigate the burden of WRMSDs.

For researchers, the study emphasizes the need for longitudinal studies to establish causal relationships between risk factors and WRMSDs. Future investigations should explore the role of behavioural and environmental factors in greater detail.

## Conclusion

In conclusion, this study highlights the high prevalence of WRMSDs among ICU nurses and identifies significant associations with factors such as longer ICU tenure, high shift frequency, increased physical workload, greater risk perception, and lack of regular exercise. The findings emphasize the urgent need for targeted interventions to address modifiable risk factors, including ergonomic training, workplace modifications, and education programs to enhance risk awareness and preventive behaviours. Qualitative insights further underscore the importance of institutional support and preventive measures, such as promoting physical exercise and risk mitigation strategies. Future research should focus on developing and evaluating comprehensive interventions that integrate physical and work environment factors to mitigate WRMSDs among ICU nurses.

## Data Availability

The datasets generated during the current study are not publicly available due to risk of compromise in privacy, but are available from the corresponding author on reasonable request.
